# Beyond Boundaries: In Search of an Integrative View on Motor Symptoms in Schizophrenia

**DOI:** 10.3389/fpsyt.2014.00145

**Published:** 2014-10-14

**Authors:** Manuel Morrens, Lise Docx, Sebastian Walther

**Affiliations:** ^1^Collaborative Antwerp Psychiatric Research Institute, University of Antwerp, Antwerp, Belgium; ^2^Psychiatric Center Alexian Brothers, Boechout, Belgium; ^3^University Hospital of Psychiatry, University of Bern, Bern, Switzerland

**Keywords:** motor, psychomotor disorders, schizophrenia, catatonia, neurological soft signs, extrapyramidal signs, assessment methods

## Motor Symptoms of Schizophrenia

Schizophrenia is typically conceived as an illness characterized by positive, negative, and cognitive symptoms. However, most schizophrenia patients also display a wide range of symptoms characterized by aberrant motor functioning. Symptoms of schizophrenia that fit this description are catatonic features, the motoric neurological soft signs (NSS), extrapyramidal symptoms (EPS), psychomotor slowing, and reduced motor activity.

### Catatonia

Catatonic symptoms form a heterogeneous group of motor, emotional, and behavioral symptoms. Mostly in line with historical reports, several recent studies demonstrate a prevalence of the catatonic features in 5–32% of schizophrenia patients (1, 2). Although the incidence of catatonia in schizophrenia may have decreased somewhat, the syndrome is still highly prevalent but often underdiagnosed (2).

### Neurological soft signs

Neurological soft signs are a heterogeneous cluster of subtle neurological signs that are generally divided into four sub-categories: sensory integration, primitive reflexes, motor coordination, and sequencing of complex motor acts, the latter two being considered “motoric” NSS (3). NSS have been demonstrated in up to 97 and 100% of neuroleptic naive and medicated first-episode patients, respectively (1).

### Psychomotor slowing

Psychomotor slowing refers to the slowing of various motor processes such as gross (e.g., gait) and fine motor (e.g., writing) movement, speech, and facial expression. Our group conducted a series of studies in which impairments in different aspects of psychomotor functioning were slowed, including planning, initiation, and execution of movements (4, 5).

Several studies assessed the amount of motor activity by means of actigraphy, which consistently demonstrated reduced motor activity levels in schizophrenia patients (2, 6). This reduction in spontaneous motor behavior was associated with negative symptom severity (7, 8).

### Extrapyramidal symptoms

Akathisia, Parkinsonism, dyskinesia, and dystonia are considered EPS. Although these clinical features are most persistently linked to antipsychotic pharmacotherapy, there is growing consensus that the use of antipsychotic medication is not the main cause but a contributing factor to motor abnormalities in schizophrenia (9). In a systematic review (10), a median rate of 9% of spontaneous dyskinesia and a median rate of 17% of spontaneous Parkinsonism was reported in antipsychotic-naive first-episode patients, thus positioning EPS as highly prevalent intrinsic symptoms of schizophrenia. Spontaneous Parkinsonism and abnormal involuntary movements are also found at increased frequencies in unaffected first degree relatives of schizophrenia patients (11).

## Positioning Motor Symptoms in Schizophrenia

When considering all motor symptoms, recent research demonstrates that 40–80% of the schizophrenic patients present with at least one categorically defined motor syndrome (12, 13). Moreover, these symptoms are associated to the patients’ social, clinical, and functional outcome (14) and can be differentiated from positive, negative, and cognitive symptoms (15). As a result, it can be argued that motor deficits deserve recognition as a fourth symptom cluster of schizophrenia.

However, despite the increasing focus on these motor symptom cluster, the positioning of the motor syndrome is hampered by some important limitations.

First, predefined motor syndromes such as catatonia, NSS, or EPS typically stem from different research traditions, and are typically investigated separately. Hence, establishing the interrelations of the different components of motor functioning as well as their associations to other symptom groups such as negative and cognitive syndromes is limited by the fact that studies investigating more than one motor symptom cluster are scarce.

Second, the different psychomotor symptom clusters are assessed with different techniques. NSS are typically assessed by use of a neurological evaluation, psychomotor slowing is consistently gauged by use of instrumental tasks such as a finger tapping test or computerized writing tasks, and catatonia and EPS are commonly appraised using clinical rating scales. As has been demonstrated, interrelations between motor symptoms are confounded by the applied assessment technique (13).

An important artifact of the existence of parallel research lines and of the boundaries imposed by the predefined symptom clusters and their assessment instruments is the conceptual overlap between the motor syndromes. For example, rigidity is a symptom assessed by EPS, catatonia, and NSS rating scales. Similarly, psychomotor slowing, clinically recognized as bradykinesia, can also be seen as a parkinsonian sign, or as a mild form of stupor (16). This overlap not only exists between the different motor syndromes, but also with cognitive and negative symptomatology. Decreased spontaneous movement is one of the features of the negative syndrome in schizophrenia. Similarly, several neuropsychological tasks that are sensitive for higher order cognitive deficits (e.g., symbol digit substitution test or trail making tasks) are often used to assess psychomotor slowing (15).

Some may argue these predefined motor symptom clusters have internal validity, since several of the symptoms of these clusters (e.g., catatonic symptoms) co-occur and these symptom clusters tend to respond to different treatment strategies: acute catatonia responds to benzodiazepines or electroconvulsive treatment; EPS rather responds to anticholinergics, although it should be noted akathisia also responds to benzodiazepines. Nevertheless, the delineation between the clusters can be questioned. Much confusion exists on which motor symptoms constitute catatonia, leading to different tools that include from only 7 up to 40 signs or symptoms. Moreover, Krüger and colleagues (17) demonstrated that catatonic presentation may vary depending on the underlying pathology, thus challenging catatonia as a homogeneous construct. Besides, as mentioned before, a strong overlap seems to exist between these motor syndromes, justifying a reappraisal of motor functioning in schizophrenia based on the deconstruction of the barriers between predefined motor symptom clusters.

Very few studies focused on the neurobiological underpinnings of motor deficits in schizophrenia, although abnormalities in both cortical (anterior cingulate cortex, supplementary motor area, premotor cortex) and subcortical (basal ganglia, thalamus) regions as well as brain stem and cerebellum have been implicated [for review, see Ref. (2)].

## Motor and Psychomotor Functions

A much more fruitful and systematic approach may be to evaluate patients based on the different domains of motor and psychomotor functions. Rainforth and colleagues (18) classified motor functioning in five main motor skill domains: positioning, mobility, manipulation, oral motor functions, and oculomotor control. Positioning refers to the ability to assume and maintain a position as well as to maintain postural control. Mobility encompasses every action that aims at traveling from one point to another (walking, climbing, crawling). Manipulation implies every interaction with your own body (e.g., clapping hands, scratching face, …) or interaction with objects (e.g., playing tennis, drawing, writing a phrase, …). Oral motor functions include speech and other oral functions such as drinking or eating. Finally, visual functions mainly encompass blinking and the ability to fix and orient a gaze.

Some of these domains (e.g., positioning) are rather basic motor skills and do not necessarily need higher order cognitive control and may mostly be executed unconsciously while other motor domains (e.g., manipulation of objects) are heavily impacted by cognitive processes involved in the planning, initiation, execution, and monitoring of these motor acts. Consequently, the functions involved in these latter motor acts are sometimes referred to as psychomotor functions. Several studies have aimed at further delineating the sub-processes involved in psychomotor functioning (4, 13, 19).

These basic motor and psychomotor functions can be affected both on a quantitative and a qualitative level; functions can be increased/reduced (quantitative) but there may also be a breach with normal motor function leading to symptoms characterized by behavior not seen in healthy patients (qualitative). A parallel comparison can be made with cognitive functioning; patients display quantitative cognitive deficits (e.g., reduced memory or attention functioning), but also qualitative cognitive abnormalities can emerge (e.g., perseveration, neologisms and, arguably, delusional thinking).

This frame of reference allows for repositioning all motor symptoms, independent of whether they are traditionally recognized as catatonic symptoms, EPS, NSS, psychomotor slowing, or diminished motor activity (see Table 1). Note that this table does not aim to exhaustively list all motor symptoms.

**Table 1:** **Proposed classification of motor symptomatology in schizophrenia**.

Function domain	Quantitative deficits	Qualitative deficits
Positioning	Posturing, rigidity	Catalepsy, tremor, tandem walk deficits
Mobility	Reduced gait, diminished movements, stupor	Manneristic walk
Manipulation	Fine motor slowing, reduced facial expression	Stereotypy, mannerisms, fine motor sequence deficits, echopraxia
Oral motor functions	Reduced speech	Orofacial dyskinesia, echolalia
Visual functions	Gaze impersistence, staring	Blepharospasms

## Assessing Motor Skills and Psychomotor Functions

An atheoretical evaluation of motor functioning in schizophrenia making use of a more broad assessment methodology (or combination of assessment techniques) that address all these motor function domains may result in a more complete appraisal of motor and psychomotor deficits in schizophrenia, both on the quantitative and qualitative level. Such an approach has the advantage of giving a general overview of all deficits and may confirm or nuance the existing classification of motor symptomatology. Their relationship with and delineation from negative and cognitive symptoms can be evaluated more properly.

Rating scales have been developed [e.g., the Rogers Scale (20)] that score symptoms from more than one motor syndrome, although these scales still tend to focus on qualitative abnormalities and to a much lesser degree on quantitative abnormalities. As a result, they do not give a complete overview of motor functioning. On the other hand, test batteries evaluating general motor functioning can be considered, e.g., the Bruininks-Oseretsky Test of motor proficiency (BOT-2) or the movement assessment battery for children (Movement ABC-2). Typically, these batteries aim to provide a comprehensive view on ones motor functioning an even generate a motor quotient, a motor equivalent of the intelligence quotient (IQ). Included tests typically assess fine and gross motor functions such as (static and dynamic) balance and precision, walking speed, force, bilateral coordination, or visuomotor control (21). Since motor skills traditionally have been viewed in relation to the normal motor development of a young child (18), these batteries have been used to assess motor deficiencies in children, typically in children with developmental disorder, or intellectual disability, but have almost no use in adults. The Bruininks motor ability test (BMAT), an adult adaptation of the BOT-2, has recently been developed, but to our knowledge has never been investigated in adult psychiatric patient samples. Nevertheless, such an instrument with a focus on broad motor functioning is more sensitive to quantitative deficits, but in its turn lacks the capacity to assess more specific qualitative deficits.

Therefore, in order to have a more complete assessment of motor deficits in schizophrenia patients (as well as patients with other psychiatric illnesses), a test battery that encompasses on one hand tests assessing the different motor functions quantitatively and on the other assessments addressing qualitative deficits is needed. A consensus on which qualitative deficits should be included and on which assessment techniques to use in such a battery will contribute to a uniformed and more complete assessment of motor functioning in psychiatric illnesses. A large study in different psychiatric populations would consequently be needed to further validate this new motor test battery.

Chen and colleagues (22) developed a comprehensive neurological evaluation scale, the Cambridge Neurological Inventory, which addresses many of the motor functions on both a quantitative and qualitative level. This interesting tool assesses different domains of motor functioning, and constitutes of three subscales: (1) Speech, eye movements, and extremity examinations, (2) Soft signs examinations, and (3) assessments of posture and movements, including catatonia and tardive dyskinesia. Sadly, after its introduction, this instrument has mostly been used for its soft signs subscale (23, 24) whereas the total scale seems to have sunk into oblivion. This scale may prove to be a good starting point to further development of the proposed wide-ranging motor test battery. In addition, the battery should make use of instrumental assessment techniques.

Given that (psycho)motor deficits have been observed in neuroleptic-naïve first-episode patients, in stabilized young patients and in chronic patients, the use of this battery would be relevant in all phases of the illness (1, 9, 13). Such a battery may be used to further investigate the neurobiological underpinnings of these deficits, as well as to differentiate intrinsic motor deficits from the antipsychotic-induced motor side effects.

## Conclusion

A wide range of motor abnormalities can be observed in schizophrenia patients and their high prevalence warrant recognition of this motor symptom cluster. However, predefined motor syndromes such as catatonia are hampered by many limitations, and a more holistic approach may be needed to further our understanding of the motor syndrome. Research focused on assessing a wide range of motor functions encompassing both basic motor skills and higher order psychomotor functions is needed. The development of a motor test battery that will both quantify the main motor functions and assess qualitative deficits may contribute to new insights in motor functioning of psychiatric patients including those suffering from schizophrenia.

## Conflict of Interest Statement

Over the last 5 years, Manuel Morrens received funding for research from Johnson & Johnson Belgium, Lundbeck Belgium and Bristol Myers Squibb Belgium and contributed to scientific activities sponsored by Astra-Zeneca Belgium, BristolMyers Squibb Belgium, Eli Lilly Belgium, Lundbeck Belgium and Johnson & Johnson Belgium. The other coauthors declare that the research was conducted in the absence of any commercial or financial relationships that could be construed as a potential conflict of interest.
